# Delayed diaphragmatic hernia after open trauma with unusual content: Case report

**DOI:** 10.1016/j.ijscr.2019.08.030

**Published:** 2019-09-04

**Authors:** Ketlen Gomes da Costa, Rafaelle Taynah Soares da Silva, Marineide Santos de Melo, Jade Thays Saldanha Pereira, Juan Eduardo Rios Rodriguez, Renato Carvalho Amaral de Souza, Izabela Augusta de Oliveira Medeiros

**Affiliations:** a Institute of Surgery of the State of Amazonas (ICEA), Travessa A, 36 – Nossa Senhora das Graças, Manaus, AM, Zip-Code: 69053-610, Brazil; b University of Amazon State Superior School of Health Sciences (UEA/ESA), Av. Carvalho Leal, 1777 - Cachoeirinha, Manaus, AM, 69065-001, Brazil; c General Surgery service at Getúlio Vargas Teaching Hospital (HUGV), Avenida Apurinã, 4 - Praça 14 de Janeiro, Manaus, AM, Zip-Code: 69020-170, Brazil; d Medical School of Federal University of Amazonas (UFAM), Rua Afonso Pena, 1053 - Praça 14 de Janeiro, Manaus, AM, Zip-Code: 69020-160, Brazil

**Keywords:** Diaphragmatic hernia, Thoracic cavity, Thoracotomy, Laparotomy, Case report

## Abstract

**Introduction:**

Post-traumatic diaphragmatic hernias are not commonly diagnosed immediately after the initial trauma. There is a greater prevalence of left hernias due to the fragility or injury in diaphragm muscle and the lack of solid and fixed structures on the left side.

**Presentation of case:**

A male patient, 30 years old, he was admitted to the emergency department presenting diffuse abdominal pain, vomiting, dyspnea, pain in the left hemithorax with worsening during forced inspiration. After performing X-ray and computed tomography (CT), the presence of organs in the abdominal cavity outside the usual position was evidenced and with discrete deviation. Immediate surgery was performed with posterolateral thoracic access through the sixth left intercostal space combined with left subcostal access. Initially, it was found jejunum, ileum and left colonic flexure and accessory spleen filling the hernial sac.

**Discussion:**

This report is the first case to report two accessory spleens in manual reduction of herniation between thoracic and abdominal cavities after trauma and percutaneous perforation. The splenectomy performed in both organs occurred due to their advanced ischemia that was due to reduced vascularity.

**Conclusion:**

The presence of the reported accessory spleen inside the thoracic cavity is only a possible variation within the possibilities in cases of diaphragmatic hernias, which does not modify the surgical procedure in a relevant way.

## Highlights

• They are classic signs of progression in the presence of a hidden lesion in the diaphragm.
• The presence of the reported accessory spleen is only a possible variation.
• The use of thoracotomy is more indicated in cases with a later diagnosis.

## 1 Introduction

Post-traumatic diaphragmatic hernias (PTDH) are not commonly diagnosed immediately after the initial trauma. Acute symptoms are uncommon, and they usually appear over time. It happens because of the negative pressure in the thoracic cavity that increases the initial injury on the diaphragm and the volume of visceral content in the thoracic cavity. This initial difficulty in the diagnosis generates serious complications that can occur through indeterminate and variable time [1,2]. These complications can be obstructive and ischemic complications. This problem is usually detected in adults due to the nature of the lesion. It facilitates the differential diagnosis of congenital hernias such as Bochdalek and Morgagni. These hernias are originated from flaws in the embryonic closure of the diaphragmatic muscle [3].

There is a greater prevalence of left hernias due to the fragility or injury in diaphragm muscle and the lack of solid and fixed structures on the left side. Both perforating and blunt trauma can cause injury. However, there is a tendency of having greater caution in cases of blunt trauma of high impact due to the kind of the trauma (mainly traffic accidents) and the initial difficulty of diagnosing diaphragmatic hernia [4,5]. The most common symptoms are dyspnea and chest pain as well as diffuse abdominal pain commonly localized in the upper abdomen, stopping the evacuation in cases more chronic or progressing to severe pain in situations of ischemia. The clinic tends to mimic haemothorax or pneumothorax and can result in poor management of closed chest drainage if there is not a careful anamnesis [6]. The conventional chest X-ray still holds the diagnosis in most cases. Despite that in cases of occulted chronic hernias, minimum size or right diaphragmatic hernias, the use of computed tomography is the best solution for evidence of injury, avoiding further complications.

Computed tomography in 3D, scintigraphy, and magnetic resonance help to identify specific organs with more accuracy. However, they should not delay the conduct in cases of urgency and are not available at all health centers. In addition of all of these factors, there are the presence of several abdominal visceral organs that may be the contents of the hernial sac, depending on the location of the trauma, such as spleen, splenic flexure, jejunum, ileum, liver (right hernia) which can lead to various complications such as pneumonia, enteric ischemia, twisting of the hilum (especially spleen) and it's perforation. Laceration of organs is mostly from perforating cause [7]. This work has been reported in line with the SCARE criteria [8].

## 2 Report

A 30 years old male patient, born and raised in the countryside of Amazonas State, was admitted to the emergency department in Manaus city. The distance between the emergency department and its location was 40 km. He had constipation for two days, diffuse abdominal pain, vomiting, dyspnea, pain in the left hemithorax with worsening during forced inspiration. He denied other clinical complaints and associated clinical comorbidities or the use of daily medications. He just reported a prior emergency surgery from nine years ago due to a stab injury in his left flank. This first trauma occurred due to perforation by steel rebar at left thoracoabdominal region, evolving with hemopneumothorax. Only chest drainage was performed at the time, as there were no signs of abdominal cavity injury or other complaints, according to the patient, and presented discharge with improvement five days later. He did not present social and demographic data worth mentioning. At the examination, the patient presented good general condition, lucid and oriented in time and space, pulmonary auscultation with reduction of vesicular murmur in the middle and basis of left hemithorax, with tympanic percussion, respiratory rate 15irpm.

**Fig. 1:**
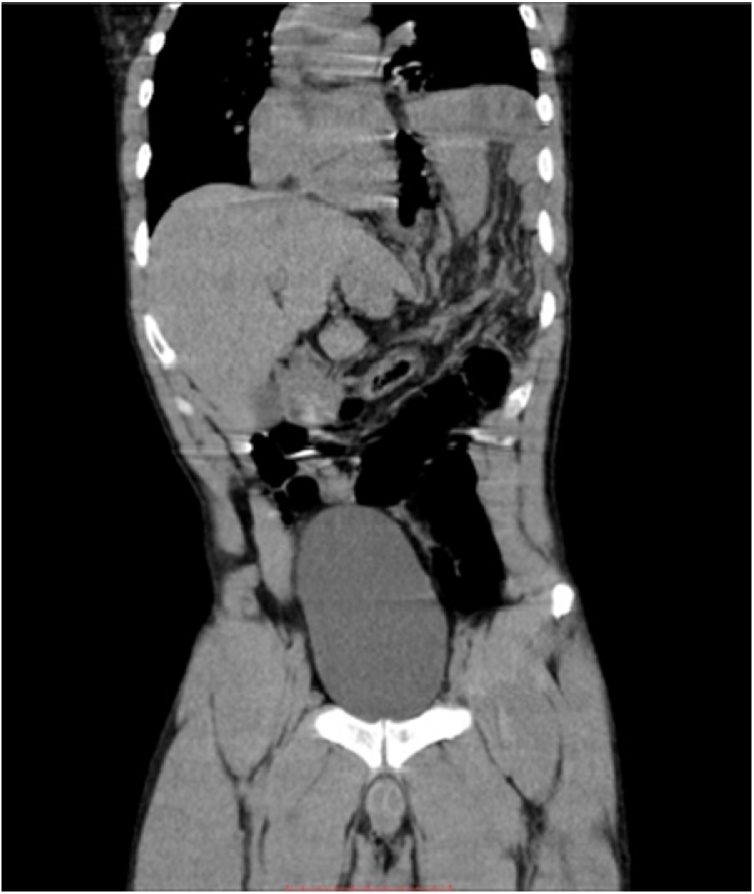
CT scan of the chest and abdomen showing voluminous left diaphragmatic hernia.

Abdomen was globose, distended, diminished breath sounds, pain to superficial and deep palpation diffusely, with higher intensity in deep palpation in left hypochondrium, percussion without alterations. After pain control with venous analgesia, chest X-ray and thorax/abdomen computed tomography were performed with evidence of abdominal material filling the left thoracic cavity on its middle portion, elevation of gastric bulge height with a contralateral deviation of the mediastinum and without evidence of pneumothorax or atelectasis. The tomography showed the hernia content, with the presence of splenic flexure of the colon, stomach, omentum, spleen and organs in the abdominal cavity outside the usual position with discrete deviation (Fig. 1).

**Fig. 2:**
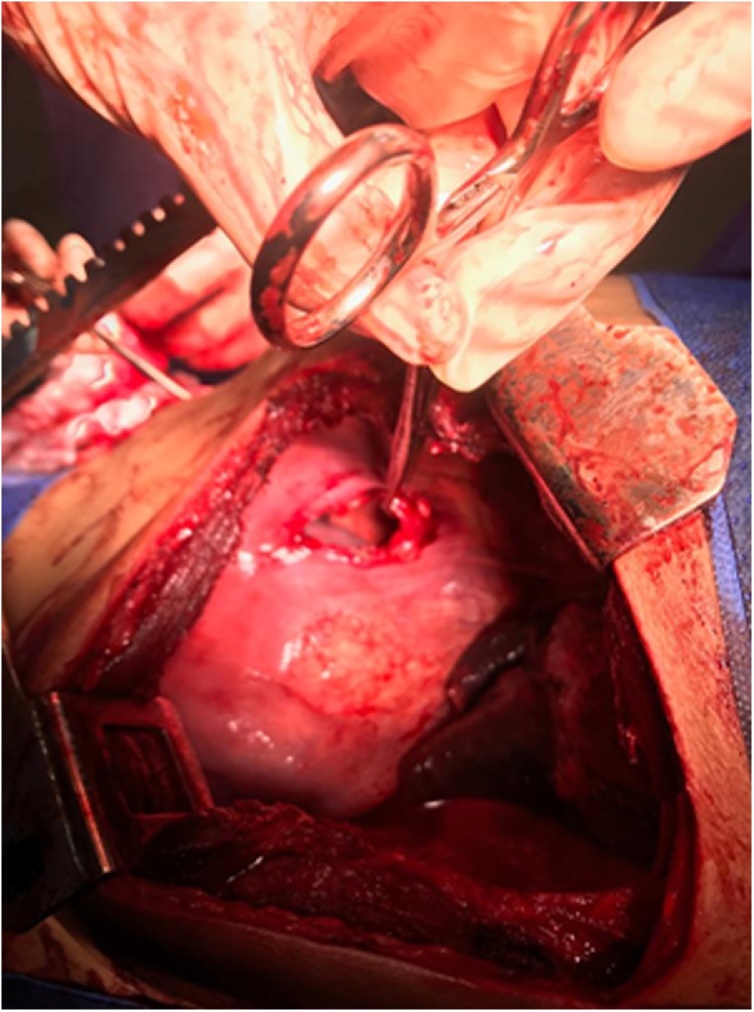
Hernia orifice in diaphragm.

**Fig. 3:**
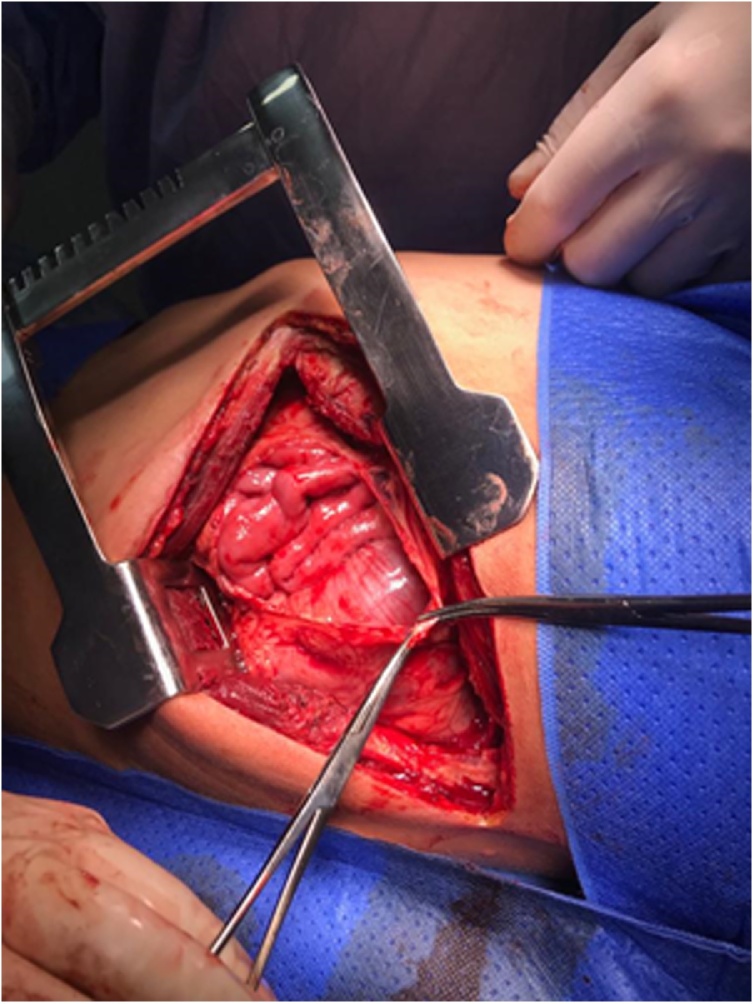
Hernia orifice seen by thoracotomy.

Immediate surgery was performed with posterolateral thoracic access on the sixth intercostal space on the left side. It was initially found jejunum and ileum filling the chest cavity, bowel with adequate blood flow, good viability, no perforations or adhesions in pleura (Fig. 2, Fig. 3). Transverse colon was in a cavity with firm adhesions in a gastrocolic ligament. Spleen was free and had unchanged vascularity, but it had two portions of similar splenic tissue with its vascularity with signs of ischemic suffering, which were characterized as accessory spleens by topography.

**Fig. 4:**
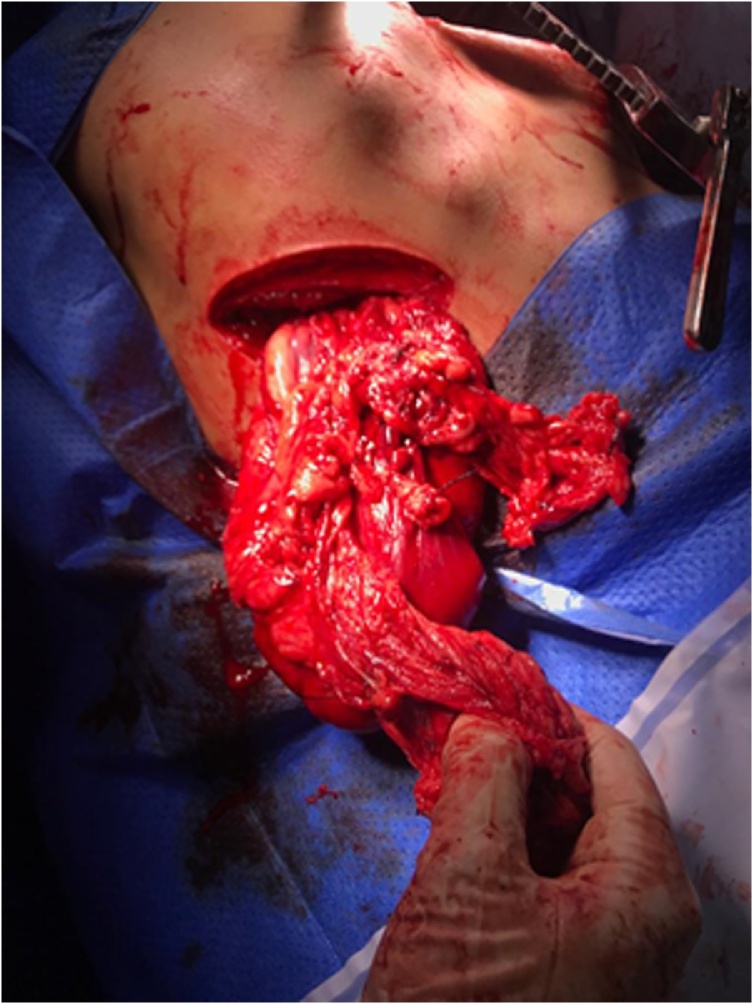
Enlargement of the hernia to reduce hernia content.

**Fig. 5:**
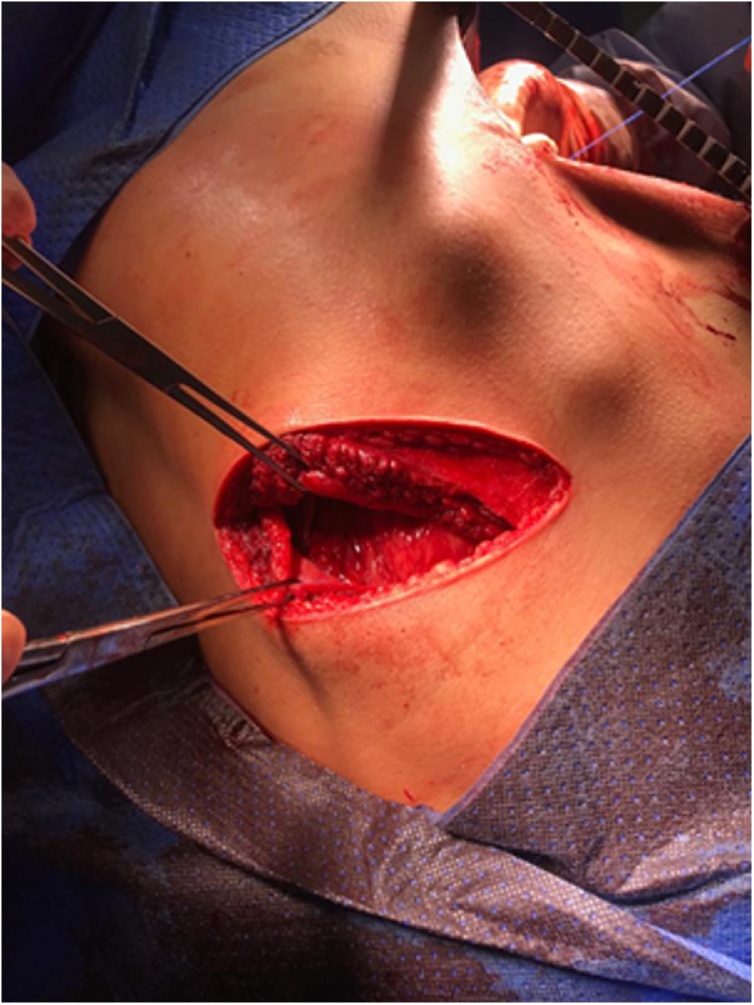
Complete reduction of the content by laparotomy with subcostal access to the left.

Lysis of adhesion herniary sac was realized and continued to decrease through the same thoracoabdominal wound. However, it was unsuccessful after consecutive attempts because of the large volume of the contents. It was chosen a concomitant left subcostal laparotomy incision to reduce the bowel to obtain a total success in reducing all hernia contents to the abdominal cavity (Fig. 4, Fig. 5). Synthesis of diaphragm injury performed with 0 polypropylene thread and single sutures in separate stitches. Finished positioning abdominal contents, secondary closed chest drainage was performed in 8° left intercostal space and synthesis by layers of surgical access incisions. After anesthetic recovery, the patient remained stable with a vesical delay probe, blood pressure at 160 × 100 mmHg, temperature 36 °C, heart rate of 80 beats per minute and 20 breaths per minute maintained under analgesia with intravenous tramadol 100 mg/2 ml and administered 1 g of intravenous cephalothin. He evolved without complications with thoracic tube withdrawal on the fifth postoperative day, stable vital signs and with a mild oral diet. He was discharged on the sixth postoperative day, asymptomatic, with referral to general surgery outpatient clinic and guidelines on surgical wound care.

## 3 Discussion

Within the natural course of hernia, the presented case was in chronic phase with association of intestinal obstruction and ischemic symptoms. They are classic signs of progression in the presence of a hidden lesion in the diaphragm. Because of the extent of the injury, the symptoms themselves were not cleared. The presented case is the first to report two accessory spleens in manual reduction of herniation between thoracic and abdominal cavities after trauma and percutaneous perforation.

In the literature, there is only one case accessory spleen in Bochdalek Syndrome and other incidental findings in asymptomatic diaphragmatic hernia unrelated to previous trauma [9,10]. The splenectomy performed in both organs occurred due to their advanced ischemia due to reduced vascularity [11]. The splenectomy was not performed in the primary spleen because of the proper flow in the splenic hilum and absence of parenchymal lesions. Despite the successful exeresis, the immunological consequences suggest being irrelevant to the evolution of the patient. Thoracic and abdominal access is commonly discussed in the current literature, but without a consensus about which technique would be the best technique. They conclude that few studies are adequately evaluating both techniques or when there is a need to perform them simultaneously, whether by the extent of diaphragmatic wound or volume of abdominal contents into the chest cavity [12].

In the reported case, the reason for using thoracotomy to undergo laparotomy was the initial difficulty in reducing the visceral organs to the original cavity. Using two surgical approaches was better because of control of the positioning of the organs and intraoperative visualization. Some literature considers the use of one incision the ideal due to the minor trauma caused by the surgery and for the shorter hospitalization time [13]. However, it is a factor that requires appropriate discussion. The use of thoracotomy is more indicated in cases with a later diagnosis because of the presence of adhesion between the loops and the pleura, while the use of laparotomy is more related to the acute condition. Probably, it happens due to the standard surgical exploration of abdominal trauma.

Diaphragm closure can also be associated with fenestrated polypropylene mesh to avoid relapses although this method of repair is referred to be the best result of techniques for correction of abdominal wall hernias, not diaphragmatic lesions [14,15]. There are not any studies about frequency of relapse in these cases, only the use of simple raffia is more common nowadays. Postoperative complications are common, especially pneumonia, but the mortality is low in most cases [16]. If there is presence of perforation in the gastrointestinal tract or hernia necrosis, the mortality increases considerably, which implies always being extra cautious when having a suspicious diagnosis [17,7].

## 4 Conclusions

The presence of the reported accessory spleen is only a possible variation within the possibilities in cases of diaphragmatic hernias, which does not modify the surgical procedure in a relevant way. Regarding the difficulty in reducing content only with thoracotomy, studies should be conducted to compare the possible techniques, indications and complications. We currently have only the consensus that thoracotomy is indicated when the hernia is late and laparotomy is most appropriate immediately after trauma, due to intraoperative characteristics and their technical difficulties. The studies should formalize systematic care and easy access to other professionals, especially to newly trained or resident surgeons.

## Funding

We do not have any funding source, this manuscript is just a case report, not a research.

## Ethical approval

As the manuscript is not a research study, we only have the patient consent for writing and others forms of publication. Also, the ethical approval for this case reports has been exempted by our institution.

## Consent

Written informed consent was obtained from the patient for publication of this case report and accompanying images. A copy of the written consent is available for review by the Editor-in-Chief of this journal on request.

## Author contribution

Marineide Melo, Jade Saldanha and Juan Rodriguez made contributions to conception and design. collected the patient details and wrote the paper. Ketlen Sousa, Rafaelle Silva made contributions to patient management. Izabela Medeiros and Renato Souza critically revised the article. All authors read and approved the final manuscript.

## Registration of research studies

The manuscript is a case report, not considered a formal research involving participants.

## Guarantor

Dra. Ketlen Gomes.

## Provenance and peer review

Not commissioned, externally peer-reviewed.

## Declaration of Competing Interest

We do not have any conflicts of interests.
